# The association between liver function tests abnormalities and type 2 diabetes mellitus patients in Saudi Arabia: a cross-sectional study

**DOI:** 10.3389/fcdhc.2025.1617641

**Published:** 2025-07-29

**Authors:** Nimer Mehyar, Ziyad Alhajeri, Muath Alosaimi, Ziyad Alanazi, Abdulkarim Alanazi, Raghib Abusaris

**Affiliations:** ^1^ College of Science and Health Professions, King Saud bin Abdulaziz University for Health Sciences (KSAU-HS), Riyadh, Saudi Arabia; ^2^ King Abdullah International Medical Research Center (KAIMRC), Riyadh, Saudi Arabia; ^3^ College of Medicine, King Saud bin Abdulaziz University for Health Sciences (KSAU-HS), Riyadh, Saudi Arabia; ^4^ College of Public Health and Health Informatics, Department of Epidemiology and Biostatistics, King Saud bin Abdulaziz University for Health Sciences, Riyadh, Saudi Arabia

**Keywords:** liver function test, type 2 diabetes mellitus, comorbidities, liraglutide, allopurinol, Saudi Arabia

## Abstract

**Introduction:**

Increasing evidence shows that hyperglycemia-induced glucotoxicity and lipotoxicity that usually accompany diabetes development damage the endoplasmic reticulum and mitochondria of the hepatocytes in diabetic patients. Clinical studies highlighted the association between type 2 diabetes mellitus, comorbidities, and medications with liver function. The objective of this study is to explore the association between liver function tests’ abnormalities and comorbidities, medications, and other risk factors in type 2 diabetes patients registered in the Best-Care system of the Saudi Ministry of National Guard-Health Affairs.

**Methods:**

This is a cross-sectional study employing a chart of patients diagnosed with type 2 diabetes mellitus. We drew a simple random sample of 523 T2DM patients who had a liver function test from the Best-Care database of the Ministry. We applied various statistical analyses, including Student’s independent t-test, Pearson’s chi-squared test, Fisher’s exact test, and odd ratios, to measure associations between different variables and liver function tests’ abnormalities.

**Results:**

About 35% of patients included in this study showed an abnormal level of gamma-glutamyl transferase and prothrombin time. Abnormalities of serum albumin, prothrombin time, and total serum protein tests were significantly associated with age (P < 0.05). Gamma-glutamyl transferase test abnormalities were significantly associated with gender (P < 0.05). The study found associations between several comorbidities and the abnormalities of liver function tests. These tests include the total bilirubin, albumin, total serum protein, gamma-glutamyl trans, international normalized ratio, and alanine aminotransferase. The associations were at significant levels (P < 0.05). Liraglutide was significantly associated with aspartate aminotransferase (OR = 14.40, 95% CI = 2.8, 73.2), while allopurinol was significantly associated with international normalized ratios (OR = 24.67, 95% CI = 2.95, 206.58) and total serum protein (OR = 5.44, 95% CI = 1.43, 20.83).

**Discussion:**

This study is the first to examine the association between type 2 diabetes mellitus and liver function tests’ abnormalities in Saudi Arabia. Although the results have a limited generalizability due to inherent biases, the findings align with similar studies in other populations. The study stresses the need to monitor liver functions, especially of T2DM patients who suffer from other conditions.

## 1 Introduction

Diabetes mellitus (DM) is a chronic condition characterized by an abnormal elevation of glucose levels in the blood, i.e., hyperglycemia (1). According to a recent International Diabetes Federation report, approximately 17.7% of the adult population in Saudi Arabia has DM (2). The Centers for Disease Control and Prevention reported a comparable prevalence in the U.S. population close to 14.7% (3). Several resources rank Saudi Arabia as having one of the highest prevalences of DM in North Africa and the Middle East (4–6). Type 2 diabetes mellitus (T2DM) is often accompanied by comorbidities such as overweight or obesity, hyperlipidemia, chronic kidney disease (CKD), and cardiovascular disease (7, 8). Growing evidence indicates that patients with DM are at higher risk of several chronic liver diseases, including non-alcoholic fatty liver disease, alcoholic cirrhosis, chronic hepatitis C, and hemochromatosis (9–14). The liver plays an essential role in glucose homeostasis by coordinating several glucose formation and utilization pathways at normal physiological levels. These pathways include glycogenesis, glycogenolysis, glycolysis, and gluconeogenesis at normal physiological levels (15). Hyperglycemia occurs when the rates of glucose formation persist to exceed the rates of glucose utilization (16). Investigators proposed that hyperglycemia-induced glucotoxicity and lipotoxicity have deleterious effects on the endoplasmic reticulum and mitochondria of the hepatocytes (17–20). They predicted that these damages could potentially trigger a series of events that lead to the death of the hepatocytes and thus elevate liver function tests (LFTs) values (10).

LFTs are typically categorized into three groups: first, liver function indicators, including prothrombin time (PT), international normalized ratio (INR), albumin (ALB), and total serum protein (TP). Second, liver injury indicators include gamma-glutamyl transferase (GGT), lactate dehydrogenase, and glutamate dehydrogenase. Third, viral hepatitis serological tests (21). Several clinical studies underlined the association of T2DM with elevated levels of alanine aminotransferase (ALT) and GGT, particularly in older individuals (22–26). Beyond the physiological effects of T2DM, pharmacological interventions further influence LFT outcomes (27). Other studies showed that insulin-sensitizing agents like metformin and pioglitazone decrease aspartate aminotransferase (AST) and ALT (28). Insulin stimulators like sulfonylureas, on the other hand, have been associated with elevated levels of ALT and GGT (22). One study showed that alpha-glucosidase inhibitors such as acarbose cause a significant rise in ALT, AST, and total bilirubin (TBIL). In some cases, this drug increased the risk of hepatotoxicity (29). Moreover, many diabetic patients take protective therapy against diabetic complications, and this can contribute to elevated LFTs. For example, statins, widely used medications by diabetics, have been associated with 5% of cases of clinical liver injury (30).

One study estimated that diabetic patients and DM-related expenditures cost Saudi Arabia about 17 billion SAR in 2013, which represented approximately 0.61% of the country’s GDP that year, estimated at 2.8 trillion SAR (31). The study projected that this cost could rise up to 27 billion SAR in the following years (32). In most cases, DM is associated with one or more risk factors and comorbidities (33). These comorbidities significantly impact the type and volume of medical health care utilization, ultimately increasing T2DM health costs (34). Additionally, several studies showed that many of these comorbidities, including cardiovascular diseases, cerebrovascular events, and CKD, contribute to liver damage (35–37). Identifying these comorbidities could enhance the development of better and less costly DM management programs that fit the different health care needs of different comorbidities’ profiles (34, 38).

The aim of this study is to examine the association between LFTs abnormalities and DM, considering risk factors, comorbidities, and medications. We used the reference values of LFTs adopted by the Best-Care system of the Saudi Ministry of National Guard-Health Affairs (MNGHA) to indicate abnormalities.

## 2 Methods

### 2.1 Study design and subjects

We conducted a cross-sectional study using chart reviews, targeting MNGHA T2DM patients who tested liver function in the hospital. We calculated the sample size using the Cochrane formula n=p(1-p) (z/m)^2, where p is the estimated proportion of abnormality, z is the critical value corresponding to the confidence level, and m is the margin of error. For p=0.5, a confidence level of 95% (z=1.96), and a margin of error of 5%, the desired sample size is 385 participants (39). It is important to notice that some records were missing information like BMI and medication. To compensate for this missing information, we collected the records of a total of 523 T2DM patients who matched our inclusion and exclusion criteria dated from April 2015 to December 2022. Our inclusion criteria included all T2DM patients with previous LFTs. The study included male and female patients with T2DM who had done an LFT in the hospital. The study excluded T2DM patients who hadn’t done an LFT before and non-diabetics with existing LFTs.

### 2.2 Data collection

We extracted a simple random sample of 523 T2DM patients from the MNGHA Best-Care database. All patients were T2DM patients and had done an LFT. We collected the last measured LFT of every sample and used it for analysis. To decide whether an LFT is normal or not, we used the reference values of the Best-Care system.

### 2.3 Statistical analysis

We used the Social Sciences Statistical Package (SPSS) version 20 to perform descriptive and inferential statistical analyses. We measured and reported the descriptive statistics parameters including the means, standard deviations, counts, value ranges, and percentages for sample characteristics, comorbidities, clinical characteristics, LFTs estimates, and medications. For the inferential statistics, we used confidence intervals to estimate the prevalence of each LFT. Then we applied independent t-test, the Pearson’s t-test, Fisher’s exact test, or odds ratios (OR) to examine the association between the sample characteristics, comorbidities, and medications with LFTs after checking the corresponding assumptions.

## 3 Results

### 3.1 Sample characteristics

To estimate the proportions of abnormal LFTs among T2DM patients with a 95% confidence interval and a 5% margin of error, the minimal sample size needed was 385 patients. However, due to the possibility of incomplete data records, we collected a simple random sample of size 531 patients from the MNGHA Best-Care database. After removing incomplete data records, we used a net of 523 patient records for analysis. The mean age of our patients is 60.62 ± 14.52 years with a range from 4 to 96 years old. Most of the patients are females (54.5%), obese (50.3%), and have no complications (88.1%). In addition, the majority use metformin 500 mg medication (59.7%), followed by metformin 1000 mg medication (29.9%). Furthermore, the vast majority (94.3%) do not use other medication; see **Table 1** below. Many T2DM patients suffered from other conditions, mainly hypertension (58.9%) and dyslipidemia (76.5%); see **Table 2** below.

**Table 1:** Sample characteristics.

Variable	Categories	Count (%)	Mean (SD)	Range
n = 523				
Age (in years)			60.62 (14.48)	4 to 96
Gender	Female	285 (54.5)		
	Male	238 (45.5)		
BMI class (n = 477)	Underweight	6 (1.3)		
	Normal	63 (13.2)		
	Overweight	168 (35.2)		
	Obese	240 (50.3)		
T2DM with complications (n = 521)	Yes	62 (11.9)		
	No	459 (88.1)		
T2DM medications (n = 395)	Metformin 500 mg	244 (61.8)		
	Metformin 1000 mg	116 (29.4)		
	Liraglutide 0.6 mg	10 (2.5)		
	Metformin 500 + Liraglutide 0.6 mg	15 (3.8)		
	Metformin 1000 + Liraglutide 0.6 mg	10 (2.5)		
Other medications	None	493 (94.3)		
	Allopurinol 100mg	18 (3.4)		
	Other(s)	12 (2.3)		

**Table 2:** Comorbidities and sample clinical characteristics.

Disease	n	Yes count (%)	No count (%)
Hypertension	421	248 (58.9%)	173 (41.1%)
Dyslipidemia	421	322 (76.5%)	99 (23.5)
Hypothyroidism	439	68 (15.5%)	371 (84.5%)
Chronic kidney disease	439	28 (6.4%)	411 (93.6%)
End-stage renal disease	439	3 (0.7%)	436 (99.3%)
Stroke	438	23 (5.3%)	415 (94.7%)
Leukemia	439	2 (0.5%)	437 (99.5%)
Lymphoma	439	1 (0.2%)	438 (99.8%)
Cancer	439	10 (2.3%)	429 (97.7%)
Liver cirrhosis	439	4 (0.9%)	435 (99.1%)
Fatty liver	439	6 (1.4%)	433 (98.6%)
Gastroesophageal reflux disease	439	12 (2.7%)	427 (97.3%)
Pulmonary embolism	439	2 (0.5%)	437 (99.5%)
Myocardial in Fraction	439	17 (3.9%)	422 (96.1%)
Angina	432	6 (1.4%)	432 (98.6%)
Peripheral vascular disease	439	3 (0.7%)	436 (99.3%)
Asthma	439	53 (12.1%)	386 (87.9%)
Chronic obstructive pulmonary disease	439	3 (0.7%)	436 (99.3%)
Heart failure	439	26 (5.9%)	413 (94.1%)

**Table 3:** Liver function tests estimates.

Test	Normal range ^a^	n	Normal count (%)	Abnormal count (%)	Abnormality 95% C. I.
Total bilirubin	3.5-20.5 μmol/L	454	427 (94.1%)	27 (5.9%)	3.77%, 8.12%
Albumin	34–48 g/L	457	432 (94.5%)	25 (5.5%)	3.39%, 7. 56%
Alkaline phosphatase	40–150 IU/L	460	440 (95.7%)	20 (4.3%)	2.48%, 6.21%
Gamma-glutamyl transferase	9–36 U/L	152	98 (64.5%)	54 (35.5%)	27.92%, 43.14%
Prothrombin time	9.38-12.34 s	126	80 (63.5%)	46 (36.5%)	28.10%, 44.91%
International normalized ratio	0.8-1.2	126	119 (94.4%)	7 (5.6%)	1.56%, 9.56%
Total protein	60–83 g/L	452	433 (95.8%)	19 (4.2%)	2.46%, 6.32%
Alanine aminotransferase	5–55 U/L	103	100 (97.1%)	3 (2.9%)	0.33%, 6.16%
Aspartate aminotransferase	5–34 U/L	392	368 (93.9%)	24 (6.1%)	3.75%, 8.50%

^a^Ranges and units are based on Best-Care Database.

### 3.2 Liver function tests

The point and interval estimate of the abnormality detected by the nine LFTs are listed below (**Table 3**). We noticed that aside from the GGT and PT tests, the abnormalities of other LFTs were small (≤ 6.1%).

### 3.3 General characteristics association with liver function tests

Student’s independent t-test revealed a significant difference in average age between the normal and abnormal groups of ALB, PT, and TP tests (p < 0.05) (**Table 4**). It is important to note that we did not calculate the OR values for age since we did not categorize the age into groups (**Supplementary Table S1**). OR values and Pearson’s chi-squared test showed a strong association between gender (female as reference) and the GGT test (p < 0.05, OR = 2.38, 95% CI = 1.21, 4.7). OR values and Fisher’s exact test showed no association between LTFs and body weight (normal weight as a reference). Similarly, OR values and Fisher’s exact test showed no association between LTFs and major complications (no complications as a reference) (**Supplementary Table S1**, **Table 4**).

**Table 4:** Sample general characteristics associated with LFTs.

Test	Category	Variable								
		Gender	Sex		Weight				MCC	
		Years (SD)	Female, n	Male, n	UW, n	NW, n	OW, n	OB, n	Yes, n	No, n
TBIL	Normal	60.94 (13.79)	236	191	4	56	145	211	374	51
	Abnormal	60.15 (16.21)	14	13	1	3	10	13	24	3
	*p*	0.774^a^	0.729^b^		0.493^c^				1^c^	
Alb	Normal	60.33 (13.67)	247	185	5	54	148	217	378	52
	Abnormal	71.56 (12.45)	11	14	1	5	8	8	21	4
	*p*	<0.001^a*^	0.196^b^		0.159^c^				0532^c^	
ALP	Normal	60.74 (13.53)	244	196	6	55	151	219	384	54
	Abnormal	63.40 (21.71)	11	9	0	7	4	7	17	3
	*p*	0.593^a^	0.968^b^		0.051^c^				0.727^c^	
GGT	Normal	60.22 (15.50)	59	39	3	21	31	37	79	18
	Abnormal	61.63 (13.59)	21	33	1	7	17	28	46	8
	*p*	0.814^a^	0.012^b*^		0.397^c^				0.559^b^	
PT	Normal	63.59 (14.31)	45	35	3	15	32	28	67	13
	Abnormal	68.80 (14.11)	26	20	0	8	14	23	35	11
	*p*	< 0.05^a*^	0.976^b^		0.292^c^				0.292^b^	
INR	Normal	64.90 (14.49)	ND	ND	3	22	45	47	95	24
	Abnormal	75.57 (8.10)	ND	ND	0	1	1	4	7	0
	*p*	0.056^a^	ND		0.630^c^				0.345^c^	
TP	Normal	60.51 (13.91)	238	195	5	54	147	217	382	49
	Abnormal	70.68 (11.08)	10	9	0	5	7	6	15	4
	*p*	0.002^a*^	0.841^b^		0.220^c^				0.262^c^	
ALT	Normal	61.51 (14.43)	53	47	2	19	32	43	81	18
	Abnormal	45.00 (9.64)	1	2	1	0	1	1	2	1
	*p*	0.052^a^	0.499^c^		0.098^c^				0.465^c^	
AST	Normal	59.97 (13.05)	213	155	4	45	134	183	324	44
	Abnormal	59.71 (16.05)	12	12	0	3	8	13	23	0
	*p*	0.925^a^	0.449^b^		0.964^c^				0.092^c^	

^a^Student’s independent t-test, ^b^Pearson’s chi-squared, ^c^Fisher’s exact test, ^*^ significant at p < 0.05, ND, not determined; NW, normal weight; OB, obese; OW, overweight; UW, underweight; MCC, major complications or comorbidities.

**Table 5:** Sample comorbidities characteristics associated with LFTs.

Test	Category	Condition											
		CKD		LC		FLD		Stroke		HF		Lymphoma	
		No, n	Yes, n	No, n	Yes, n	No, n	Yes, n	No, n	Yes, n	No, n	Yes, n	No, n	Yes, n
TBIL	Normal	349	24	371	2	367	6	353	20	350	23	372	1
	Abnormal	20	3	21	2	23	0	20	3	21	2	23	0
	*p*	0.200^a^		0.018^a*^		1^a^		0.141^a^		0.648^a^		1^a^	
Alb	Normal	356	21	373	4	371	6	358	18	356	21	376	1
	Abnormal	13	4	17	0	17	0	13	4	14	3	17	0
	*p*	0.017^a*^		1^a^		1^a^		0.011^a*^		0.077^a^		1^a^	
ALP	Normal	361	25	383	3	381	5	365	21	363	23	385	1
	Abnormal	12	2	13	1	13	1	12	2	12	2	14	0
	*p*	0.242^a^		0.133^a^		0.194^a^		0.189^a^		0.215^a^		1^a^	
GGT	Normal	78	5	83	0	83	0	79	4	80	3	83	0
	Abnormal	41	6	45	2	45	2	40	7	40	7	47	0
	*p*	0.204^a^		0.129^a^		0.129^a^		0.096^a^		0.035^a*^		ND	
PT	Normal	59	12	70	1	69	2	64	7	63	8	70	1
	Abnormal	32	7	38	1	39	0	31	8	31	8	39	0
	*p*	0.889^b^		1^a^		0.538^a^		0.119^b^		0.188^b^		1^a^	
INR	Normal	88	16	103	1	102	2	90	14	91	13	103	1
	Abnormal	3	3	5	1	6	0	5	1	3	3	6	0
	*p*	0.063^a^		0.107^a^		1^a^		1^a^		0.039^a*^		1^a^	
TP	Normal	355	24	376	3	373	6	359	20	356	23	379	0
	Abnormal	12	3	14	1	15	0	12	3	13	2	14	1
	*p*	0.075^a^		0.144^a^		1^a^		0.050^a*^		0.245^a^		0.038^a*^	
ALT	Normal	87	3	89	1	90	0	84	6	83	7	90	90
	Abnormal	3	0	3	0	2	1	3	0	3	0	3	3
	*p*	1^a^		1^a^		0.032^a*^		1^a^		1^a^		ND	
AST	Normal	310	22	329	3	326	6	315	17	313	19	331	1
	Abnormal	22	2	24	0	24	0	22	2	20	4	24	0
	*p*	0.671^a^		1^a^		1^a^		0.372^a^		0.059^a^		1^a^	

^a^ Fisher’s exact test, ^b^ Pearson’s chi-squared test, ^*^ significant at p < 0.05, ND, not determined; CKD, Chronic Kidney Disease; FLD, Fatty Liver Disease; HF, Heart Failure; LC, Liver Cirrhosis.

### 3.4 Comorbidities association with liver function tests

In order to enhance the clarity of the presented results and avoid confusion, we categorized the observed associations between comorbidities and LFT’s into four groups based on their statistical significance. These categories are: associations supported by both OR and hypothesis tests like t-test, Pearson’s chi-squared test, or Fisher’s exact test; associations suggested by hypothesis tests but not quantified by odds ratios due to indeterminate values; associations suggested by odds ratios but not statistically significant when examined by hypothesis tests; and cases where no statistically significant associations were detected.

#### 3.4.1 Associations supported by odds ratios and hypothesis tests

We observed a significant association between liver cirrhosis (LC) and the TBIL test (p < 0.05, OR = 17.67, 95% CI = 2.38, 131.69). We also found that stroke and CKD were significantly associated with the ALB test (p < 0.05, OR = 5.22, 95% CI = 1.57, 17.39; p < 0.05, OR = 6.12, 95% CI = 1.82, 20.66). In addition, heart failure (HF) is significantly associated with both the GGT and INR tests (p < 0.05, OR = 4.67, 95% CI = 1.15, 19.02; p < 0.05, OR = 7.00, 95% CI = 1.28, 38.42). Stroke was significantly associated with the TP test (p < 0.05, OR = 4.49, 95% CI = 1.18, 17.19) (**Table 5**; **Supplementary Table S2**).

#### 3.4.2 Associations indicated by hypothesis tests but not quantified by odds ratios

In some cases, Fisher’s exact test revealed significant associations between certain comorbidities and LFTs (p < 0.05). Due to the small number of cases, ORs could not be reliably calculated, and associations could not be statistically confirmed. We observed this outcome in the case of lymphoma association with TP and the association of fatty liver disease (FLD) with the ALT test (**Table 5**; **Supplementary Table S2**).

#### 3.4.3 Associations indicated by odds ratios but not statistically significant by hypothesis tests

In other cases, the OR values indicated associations between comorbidities and LFTs. However, Fisher’s exact and Pearson’s chi-squared tests did not confirm these associations (p > 0.05). We observed this outcome in the associations of CKD with INR (p = 0.063, OR = 5.50, 95% CI = 1.02, 29.71), LC with INR (p = 0.107, OR = 20.60, 95% CI = 1.12, 379.5), heart failure (HF) with AST (p = 0.059, OR = 3.30, 95% CI = 1.03, 10.61), end-stage renal disease (ESRD) with ALB (p = 0.124, OR = 11.72, 95% CI = 91.01, 136.090), ESRD with alkaline phosphatase (ALP) (p = 0.102, OR = 14.77, 95% CI = 91.26, 173.450), ESRD with TP (p = 0.110, OR = 13.46, 95% CI = 91.16, 157.450), pulmonary embolism (PE) with TBIL (p = 0.113, OR = 16.91, 95% CI = 1.03, 279.47), PE with TP (p = 0.075, OR = 27.00, 95% CI = 1.61, 454.21), leukemia with TP (p = 0.075, OR = 27.00, 95% CI = 1.61, 454.21), chronic obstructive pulmonary disease (COPD) with INR (p = 0.107, OR = 20.60, 95% CI = 1.12, 379.5), myocardial infarction (MI) with TBIL (p = 0.058, OR = 4.15, 95% CI = 1.1, 15.77), and MI with AST (p = 0.060, OR = 4.17, 95% CI = 1.08, 16.1) (**Supplementary Tables S2**, **S3**). It is important to note that some of the previously presented CIs are implausibly wide, likely due to small sample sizes. This limits the interpretability of these associations and increases their uncertainty. For that reason, we did not consider these associations significant.

#### 3.4.4 No statistically significant associations detected

Finally, we did not observe any association between LFT abnormalities and hypertension, dyslipidemia, hypothyroidism, gastroesophageal reflux, peripheral vascular disease, cancer, and angina (**Supplementary Table S3**).

### 3.5 Medication association with liver function tests

Patients received one of five types of treatments for T2DM: 500 mg metformin per day, 1000 mg of metformin daily, 0.6 mg of liraglutide daily, 500 mg of metformin plus 0.6 mg of liraglutide daily, or 1000 mg of metformin plus 0.6 mg of liraglutide daily. Fisher’s exact test revealed that T2DM medications were significantly associated with the AST test. Calculated OR values of each treatment using the 500 mg metformin as a reference revealed that the 0.6 mg liraglutide per day treatment is significantly associated with AST (OR = 14.40, 95% CI = 2.84, 73.23). We also used Fisher’s exact test to measure the association between LFT and non-diabetes treatments. These treatments include allopurinol, amiodarone, amoxicillin/clavulanate, and carbamazepine. Fisher’s exact test revealed that non-diabetes medications were significantly associated with the INR and TP tests. Calculated OR values of these treatments using “no treatment” as reference revealed that 100 mg of allopurinol per day treatment has a significant association with INR (OR = 14.80, 95% CI = 2.01, 109.48) and TP (OR = 5.44, 95% CI = 1.43, 20.83). In addition, the OR value of the group of patients who received non-diabetes treatments other than allopurinol revealed an association with INR (OR = 24.67, 95% CI = 2.95, 206.58) (**Table 6**, **Supplementary Table S4**).

**Table 6:** Sample medication characteristics associated with LFTs.

Test	Category	T2DM medication					Other conditions medications		
		MET 500 mg	MET 1000 mg	LIR 0.6 mg	MET 500 mg + LIR 0.6 mg	MET 1000 mg + LIR 0.6 mg	None	ALLO 100mg	Other ^b^
TBIL	Normal	211	105	10	14	10	402	16	9
	Abnormal	16	5	0	0	0	23	2	2
	*p*	0.874^a^					0.07^a^		
Alb	Normal	220	102	9	12	10	406	15	11
	Abnormal	8	5	1	2	0	22	3	0
	*p*	0.219^a^					0.1^a^		
ALP	Normal	219	111	10	14	10	412	17	11
	Abnormal	8	2	0	0	0	18	1	1
	*p*	0.813^a^					0.377^a^		
GGT	Normal	41	24	2	5	3	89	5	4
	Abnormal	22	14	2	1	0	49	4	1
	*p*	0.674^a^					0.666^a^		
PT	Normal	32	20	3	6	2	89	5	4
	Abnormal	20	13	0	2	1	49	4	1
	*p*	0.796^a^					0.666^a^		
INR	Normal	51	29	3	8	3	111	5	3
	Abnormal	1	4	0	0	0	3	2	2
	*p*	0.287^a^					0.002^a*^		
TP	Normal	218	107	10	13	9	408	15	10
	Abnormal	8	3	0	1	0	15	3	1
	*p*	0.752^a^					0.033^a*^		
ALT	Normal	47	26	3	4	1	90	5	5
	Abnormal	1	1	0	1	0	3	0	0
	*p*	0.323^a^					1^a^		
AST	Normal	192	99	4	13	8	346	16	6
	Abnormal	10	5	3	1	1	22	1	1
	*p*	0.017^a*^					0.406^a^		

^a^Fisher’s exact test, ^b^Amiodarone, amoxicillin/clavulanate, and carbamazepine, ^*^significant at p < 0.05.

MET, Metformin; LIR, Liraglutide; ALLO, Allopurinol.

## 4 Discussion

### 4.1 Principal findings

This study investigated the relationship between LFTs and DM and their association with various risk factors, including age, sex, obesity, comorbidities, and medications in patients diagnosed with T2DM who had done LFT in the MNGHA hospitals. T2DM patients who suffer from CKD, FLD, stroke, HF, LC, or lymphoma have at least one abnormal liver test. Metformin treatment of T2DM is significantly associated with abnormal AST levels.

Almost 35.5% of T2DM patients in this study showed an abnormal GGT test. This finding is consistent with multiple previous studies that emphasized the association between hyperglycemia and T2DM and high GGT levels (13, 22–26). Multiple studies confirmed GGT association with T2DM in different ethnic populations (40–47). Despite this pronounced association, there is no clear biological mechanism that explains the involvement of GGT in DM development (48–50). Some researchers speculated that GGT, a marker for hepatic steatosis, is also an indicator of insulin resistance during DM development. The inverse relationship between GGT and insulin sensitivity supports this speculation (51–53). Other researchers stressed the association between GGT and fat accumulation in the liver. Liver fat accumulation is associated with insulin resistance and T2DM development. The increased GGT levels in non-alcoholic fatty liver disease patients support this prediction (51, 54, 55). The relationship between GGT and oxidative stress can also explain the association between increased GGT levels and T2DM development (49, 56). The increased levels of GGT usually indicate an increase in cellular oxidative stress (57, 58). The pancreatic insulin-producing cells, beta cells, have less efficient antioxidant enzymes (59, 60). For that reason, they are more vulnerable to oxidative stress damages, which could ultimately compromise their ability to secrete insulin (61, 62). On the other hand, researchers noticed that T2DM male patients have significantly more GGT abnormalities compared to female patients. A previous study found that normal levels of GGT of males between the ages of 50 and 70 years old are usually higher than the normal levels of GGT in females of the same age group (63). In relation to the findings of the previous study, the normal GGT range in MNGHA Best Care is 9–36 U/L. Taking these sex-related differences into account, we can reasonably predict that more males have GGT levels closer to the upper limit. Those males are more likely to fill in the abnormal level category.

Approximately 36.5% of the T2DM patients of the current study had an abnormal PT test. These results align well with previous studies that reported shortened PT tests in DM patients (64–68). PT abnormalities usually occur due to the impaired balance between fibrinolysis and coagulation processes. DM promotes a procoagulant state. This state is characterized by a significant increase of hypofibrinolysis, activating coagulation factors and platelet activation (64, 65, 69). Hyperglycemia triggers prothrombin synthesis in the liver, which in turn increases thrombin levels and thus prompts a procoagulant state in DM patients (66, 67). Nevertheless, several studies showed no association between DM and PT, while other studies reported prolonged PT in DM patients (65, 70–73). Furthermore, the current study reports an increased PT abnormality in T2DM patients at the age of 71 or more. Admittedly, it is difficult to take into account all the possible factors that could lead to such an observation; however, one of the possible factors that could contribute to this effect is the consumption of anticoagulant medications. Using these medications, a common practice in this age group, could prolong the PT (67, 74). This may explain the significant association of PT abnormalities and older T2DM patients. The current study also reports an increase in ALB and TP abnormalities in older T2DM patients. The impairment of serum protein biosynthesis as a result of aging and DM could explain the elevated ALB and TP abnormalities (75). Previous studies showed a significant decrease in ALB and TP synthesis with aging (76, 77). Other studies showed that DM development could potentially impair serum ALB biosynthesis (78, 79).

The current study investigated the association between LFTs and T2DM and their relation to accompanying diseases. T2DM patients diagnosed with comorbidities, specifically CKD, FLD, stroke, HF, LC, or lymphoma, are more likely to have at least one abnormal LFT. T2DM patients diagnosed with LC tend to have higher TBIL test abnormalities. LC is typically associated with significantly higher TBIL concentrations than the normal level (80–82). However, several studies reported significantly lower TBIL concentrations in pre-DM and new-onset DM than T2DM (83–85). Despite DM’s apparent ameliorating effect on TBIL, this effect seems unable to overcome the increasing abnormality level of TBIL due to LC damages (82). T2DM patients suffering strokes or diagnosed with CKD seem to maintain higher ALB test abnormalities. Previous studies indicated that low serum ALB levels are associated with stroke (86–88). Other studies indicate that serum ALB is commonly lower in CKD patients. These studies suggest that lower serum ALB is one of the indicators of poor renal function (89, 90). T2DM can amplify these risks since it impairs the biosynthesis of serum proteins as previously mentioned (76, 77, 90). The current study also investigated TP and found it to be higher in T2DM patients diagnosed with stroke and lymphoma. A previous study indicated that low serum TP concentrations are associated with stroke. The same study showed that serum protein concentrations increased due to improved nutrition are associated with better stroke outcomes (91, 92). Other researchers attributed the elevated TP test abnormalities in T2DM patients suffering from lymphoma to the overproduction of immunoglobulins (93, 94). The overproduction of these proteins could potentially increase TP in lymphoma patients. The current study also revealed an association between GGT and INR test abnormalities and T2DM patients diagnosed with HF. Several studies indicated the association of high GGT levels and HF (95–99). Many of these studies focused on the association of elevated GGT and oxidative stress and its involvement in glutathione metabolism. Oxidative stress and inflammation are known risk factors for HF and other cardiovascular diseases. INR studies, on the other hand, indicated an inverse correlation between INR levels and HF and other thrombotic events (100, 101). These studies offer a partial explanation of the association between elevated GGT and INR abnormalities and HF in T2DM patients. The current study found that T2DM patients diagnosed with FLD have higher ALT activity abnormalities. These results are in agreement with several studies that indicated a strong correlation between higher ALT activity and FLD (102–105). Several researchers agree that ALT indicates liver cell damage that non-alcoholic fatty liver disease and other conditions cause with high reliability (106–108).

The majority of patients included in the current study are using metformin 500 mg alone for T2DM treatment. The remaining patients either used a higher dosage of metformin, metformin in combination with liraglutide, or liraglutide alone. We observed no association between T2DM medications and LFTs abnormalities except for their effect on the AST test. Generally, metformin is a safe drug that has low toxic effects on the hepatocytes (109, 110). There is no clear effect of metformin or liraglutide on most of the LFTs (111, 112). However, patients who used metformin 500 mg alone had significantly lower AST test abnormalities when compared to patients who received different treatment plans. The observed effect of metformin on AST agrees with several studies that indicated that long-term use of metformin reduces transaminase activities (110, 113, 114). Animal-based model studies showed that metformin treatment prevented or reversed liver cell steatosis. In these models, metformin treatment decreased serum ALT, AST, and ALP levels (115–118). These studies predicted that metformin inhibition of mitochondrial oxidative stress action mediates its effect on liver enzyme synthesis (119). About 5.7% of patients used medications other than those for T2DM. The current study showed that patients receiving medications other than those for T2DM have higher INR and TP test abnormalities. Previous studies showed that allopurinol, amiodarone, and amoxicillin/clavulanate may prolong INR and increase the risk of bleeding (120–123). One study showed that carbamazepine lowers INR and increases the risk of clotting (124). As for the TP test, there is evidence that some of these drugs, like amiodarone and carbamazepine, can bind to serum proteins (125, 126); however, it is not clear how these medications could affect these proteins synthesis and concentrations.

When considering the relatively high prevalence of T2DM in Saudi Arabia, in addition to the significant associations between LFT abnormalities and T2DM revealed in this study, the researchers recommended integrating routine monitoring of liver function into the clinical management of T2DM patients in the health care system of Saudi Arabia. Priority should be given to GGT, PT, AST, ALB, and TP tests. This protocol could improve patient care by early detection of potential liver dysfunction in T2DM patients and reduce the risk of developing severe liver-related complications.

### 4.2 Strengths and limitations

This study is the first to explore the association between T2DM and LFTs in Saudi Arabia. The access to the MNGHA Best-Care database ensured a robust and wide utilization of large and full data sets that extend over longer periods of time and spread over a wide geographical location throughout Saudi Arabia. This large data set allowed for proper sampling and statistical analyses, which in turn strengthened the validity of the drawn conclusions. Overall, the findings of this study agree with the findings of similar studies investigating other ethnicities and geographical locations; however, there are many factors that could limit the generalizability of the current study. Despite the large size of the MNGHA Best-Care database, some of the records in this study were incomplete. Moreover, this investigation restricted the sample to T2DM patients who had LFT. It is important to highlight that the exclusion of T2DM patients with untested LFTs could cause a selection bias. The current study estimated the effect of many confounding variables; however, it did not consider the effects of residual confounding factors that could affect LFTs (e.g., dietary habits, physical activity, medication adherence). It is important to acknowledge that not taking these factors into account could affect the generalizability of the findings of the current investigation. This observation reveals the need for intervention studies to investigate the association between T2DM and LFT abnormalities while minimizing the effect of confounding factors to a minimum. It is also important to notice that association analysis of LFTs and comorbidities did not adjust for potential confounders such as age, BMI, gender, and diabetes duration. These variables may influence both comorbidities and LFTs abnormalities. Multivariable analysis can give a better understanding of the effect of these factors. Finally, this study used Fisher’s exact test and Pearson’s chi-squared test to infer the significance of observed associations. The study also used the OR method to measure the significance of the associations. In most cases, there was an agreement between the OR method and other methods. However, the OR method showed additional significant associations not detected by other methods (**Supplementary Tables S1**–**S4**). We attribute this apparent discrepancy between the different types of association analysis to the very low values of terms used to calculate ORs. These low values could inflate the OR values and widen their confidence interval, which in turn would increase their uncertainty. In these cases, we only relied on the more accurate Fisher’s exact test and Pearson’s chi-squared tests to judge the associations of these variables. Taking a large sample size could potentially resolve these inconsistencies.

## 5 Conclusions

This study examines the association between T2DM and LFTs abnormalities in Saudi Arabia. The study emphasizes the positive association between T2DM and the incidence of GGT and PT test abnormalities. It reveals the association between T2DM and comorbidities, including CKD, LC, FLD, stroke, HF, and lymphoma, and several LFT abnormalities. Finally, it stresses the association between T2DM and other conditions, medications, and some LFT abnormalities. The findings of this study are in agreement with similar studies investigating various populations. However, the generalizability of the study remains limited due to intrinsic biases and uncontrolled factors. More controlled intervention studies could provide better evidence on these associations. The outcome of this study stresses the importance of monitoring the liver function of T2DM patients, especially those who suffer other comorbidities and consume additional medications besides the traditional T2DM medications.

## Data Availability

The data analyzed in this study is subject to the following licenses/restrictions: The datasets generated and/or analyzed during the current study are not publicly available due KAIMRC policies but are available from the corresponding author on reasonable request. Requests to access these datasets should be directed to mehyarn@ksau-hs.edu.sa.
