# Supplementary material for: The association between liver function tests abnormalities and type 2 diabetes mellitus patients in Saudi Arabia: a cross-sectional study
Source: Front Clin Diabetes Healthc. 2025 Jul 29;6:1617641. doi: 10.3389/fcdhc.2025.1617641 (PMC12339336; doi:10.3389/fcdhc.2025.1617641)
Supplement: Supplementary file 1 [file DataSheet1.docx]

Supplementary Material

| **Table S1: ORs of sample general characteristics associated with liver function tests** | | | | | | | | | | |
| --- | --- | --- | --- | --- | --- | --- | --- | --- | --- | --- |
| Test |  | Variable | | | | | | | | |
|  |  | Age | Gender | | Weight | | | | MCC | |
|  |  | Years (SD) | Female, n ^a^ | Male, n | UW, n | NW, n ^b^ | OW, n | OB, n | Yes, n | No, n |
| TBIL | Normal | 60.94 (13.79) | 236 | 191 | 4 | 56 | 145 | 211 | 374 | 51 |
|  | Abnormal | 60.15 (16.21) | 14 | 13 | 1 | 3 | 10 | 13 | 24 | 3 |
|  | OR (95% CI) | ND | 1.00 (0.47,2.15) | 1.15 (0.53,2.5) | 4.67 (0.4,55.74) | 1.00 (0.2,5.17) | 1.29 (0.35,4.86) | 1.16 (0.32,4.18) | 1.10 (0.32,3.76) | |
| Alb | Normal | 60.33 (13.67) | 247 | 185 | 5 | 54 | 148 | 217 | 378 | 52 |
|  | Abnormal | 71.56 (12.45) | 11 | 14 | 1 | 5 | 8 | 8 | 21 | 4 |
|  | OR (95% CI) | ND | 1.00 (0.43,2.35) | 1.70 (0.76,3.83) | 2.16 (0.21,22.3) | 1.00 (0.28,3.66) | 0.59 (0.19,1.87) | 0.40 (0.13,1.27) | 0.73 (0.24,2.19) | |
| ALP | Normal | 60.74 (13.53) | 244 | 196 | 6 | 55 | 151 | 219 | 384 | 54 |
|  | Abnormal | 63.40 (21.71) | 11 | 9 | 0 | 7 | 4 | 7 | 17 | 3 |
|  | OR (95% CI) | ND | 1.00 (0.43,2.35) | 1.02 (0.42,2.51) | ND | 1.00 (0.33,3.05) | 0.21 (0.06,0.74) | 0.26 (0.09,0.75) | 0.80 (0.23,2.81) | |
| GGT | Normal | 60.22 (15.50) | 59 | 39 | 3 | 21 | 31 | 37 | 79 | 18 |
|  | Abnormal | 61.63 (13.59) | 21 | 33 | 1 | 7 | 17 | 28 | 46 | 8 |
|  | OR (95% CI) | ND | 1.00 (0.5,2.03) | 2.38 (1.21,4.7) | 1.00 (0.09,11.24) | 1.00 (0.3,3.36) | 1.65 (0.59,4.66) | 2.28 (0.85,6.09) | 1.32 (0.53,3.26) | |
| PT | Normal | 63.59 (14.31) | 45 | 35 | 3 | 15 | 32 | 28 | 67 | 13 |
|  | Abnormal | 68.80 (14.11) | 26 | 20 | 0 | 8 | 14 | 23 | 35 | 11 |
|  | OR (95% CI) | ND | 1.00 (0.51,1.98) | 0.99 (0.48,2.06) | ND | 1.00 (0.3,3.37) | 0.83 (0.29,2.38) | 1.55 (0.56,4.28) | 0.62 (0.26,1.53) | |
| INR | Normal | 64.90 (14.49) | ND | ND | 3 | 22 | 45 | 47 | 95 | 24 |
|  | Abnormal | 75.57 (8.10) | ND |  | 0 | 1 | 1 | 4 | 7 | 0 |
|  | OR (95% CI) | ND | ND | ND | ND | 1.00 (0.06,17.02) | 0.49 (0.03,8.19) | 1.88 (0.2,17.75) | ND | |
| TP | Normal | 60.51 (13.91) | 238 | 195 | 5 | 54 | 147 | 217 | 382 | 49 |
|  | Abnormal | 70.68 (11.08) | 10 | 9 | 0 | 5 | 7 | 6 | 15 | 4 |
|  | OR (95% CI) | ND | 1.00 (0.41,2.45) | 1.10 (0.44,2.76) | ND | 1.00 (0.28,3.66) | 0.52 (0.16,1.69) | 0.30 (0.09,1.02) | 0.49 (0.16,1.51) | |
| ALT | Normal | 61.51 (14.43) | 53 | 47 | 2 | 19 | 32 | 43 | 81 | 18 |
|  | Abnormal | 45.00 (9.64) | 1 | 2 | 1 | 0 | 1 | 1 | 2 | 1 |
|  | OR (95% CI) | ND | 1.00 (0.07,16.41) | 2.26 (0.2,25.68) | ND | ND | ND | ND | 0.45 (0.04,5.18) | |
| AST | Normal | 59.97 (13.05) | 213 | 155 | 4 | 45 | 134 | 183 | 324 | 44 |
|  | Abnormal | 59.71 (16.05) | 12 | 12 | 0 | 3 | 8 | 13 | 23 | 0 |
|  | OR (95% CI) | ND | 1.00 (0.44,2.28) | 1.38 (0.61,3.15) | ND | 1.00 (0.2,5.23) | 0.90 (0.23,3.53) | 1.07 (0.3,3.9) | ND | |
| NW: normal weight, OB: obese, OW: overweight, UW: underweight, MCC: major complications or comorbidities, ND: not determined  ^a^ Females were used as reference to calculate OR, b: NW was used as reference to calculate OR, Bold: significant confidence intervals | | | | | | | | | | |

| **Table S2: ORs of comorbidities characteristics associated with liver function tests** | | | | | | | | | | | | | |
| --- | --- | --- | --- | --- | --- | --- | --- | --- | --- | --- | --- | --- | --- |
| Test |  | Condition | | | | | | | | | | | |
|  |  | CKD | | LC | | FLD | | Stroke | | HF | | Lymphoma | |
|  |  | No, n | Yes, n | No, n | Yes, n | No, n | Yes, n | No, n | Yes, n | No, n | Yes, n | No, n | Yes, n |
| TBIL | Normal | 349 | 24 | 371 | 2 | 367 | 6 | 353 | 20 | 350 | 23 | 372 | 1 |
|  | Abnormal | 20 | 3 | 21 | 2 | 23 | 0 | 20 | 3 | 21 | 2 | 23 | 0 |
|  | OR (95% CI) | 2.19 (0.61,7.87) | | **17.67 (2.38,131.69)** | | ND | | 2.65 (0.73,9.67) | | 1.45 (0.32,6.57) | | ND | |
| Alb | Normal | 356 | 21 | 373 | 4 | 371 | 6 | 358 | 18 | 356 | 21 | 376 | 1 |
|  | Abnormal | 13 | 4 | 17 | 0 | 17 | 0 | 13 | 4 | 14 | 3 | 17 | 0 |
|  | OR (95% CI) | **5.22 (1.57,17.39)** | | ND | | ND | | **6.12 (1.82,20.66)** | | 3.64 (0.97,13.64) | | ND | |
| ALP | Normal | 361 | 25 | 383 | 3 | 381 | 5 | 365 | 21 | 363 | 23 | 385 | 1 |
|  | Abnormal | 12 | 2 | 13 | 1 | 13 | 1 | 12 | 2 | 12 | 2 | 14 | 0 |
|  | OR (95% CI) | 2.41 (0.52,11.35) | | 9.83 (0.96,100.91) | | 5.87 (0.64,53.82) | | 2.90 (0.61,13.79) | | 2.64 (0.56,12.46) | | ND | |
| GGT | Normal | 78 | 5 | 83 | 0 | 83 | 0 | 79 | 4 | 80 | 3 | 83 | 0 |
|  | Abnormal | 41 | 6 | 45 | 2 | 45 | 2 | 40 | 7 | 40 | 7 | 47 | 0 |
|  | OR (95% CI) | 2.29 (0.66,7.94) | | ND | | ND | | 3.46 (0.96,12.51) | | **4.67 (1.15,19.02)** | | ND | |
| PT | Normal | 59 | 12 | 70 | 1 | 69 | 2 | 64 | 7 | 63 | 8 | 70 | 1 |
|  | Abnormal | 32 | 7 | 38 | 1 | 39 | 0 | 31 | 8 | 31 | 8 | 39 | 0 |
|  | OR (95% CI) | 1.08 (0.39,3.01) | | 1.85 (0.12,30.29) | | ND | | 2.36 (0.79,7.1) | | 2.04 (0.7,5.93) | | ND | |
| INR | Normal | 88 | 16 | 103 | 1 | 102 | 2 | 90 | 14 | 91 | 13 | 103 | 1 |
|  | Abnormal | 3 | 3 | 5 | 1 | 6 | 0 | 5 | 1 | 3 | 3 | 6 | 0 |
|  | OR (95% CI) | **5.50 (1.02,29.71)** | | **20.60 (1.12,379.5)** | | ND | | 1.29 (0.14,11.84) | | **7.00 (1.28,38.42)** | | ND | |
| TP | Normal | 355 | 24 | 376 | 3 | 373 | 6 | 359 | 20 | 356 | 23 | 379 | 0 |
|  | Abnormal | 12 | 3 | 14 | 1 | 15 | 0 | 12 | 3 | 13 | 2 | 14 | 1 |
|  | OR (95% CI) | 3.70 (0.98,14) | | 8.96 (0.88,91.58) | | ND | | **4.49 (1.18,17.19)** | | 2.39 (0.51,11.19) | | ND | |
| ALT | Normal | 87 | 3 | 89 | 1 | 90 | 0 | 84 | 6 | 83 | 7 | 90 | 90 |
|  | Abnormal | 3 | 0 | 3 | 0 | 2 | 1 | 3 | 0 | 3 | 0 | 3 | 3 |
|  | OR (95% CI) | ND | | ND | | ND | | ND | | ND | | 1.00(0.2,5.09) | |
| AST | Normal | 310 | 22 | 329 | 3 | 326 | 6 | 315 | 17 | 313 | 19 | 331 | 1 |
|  | Abnormal | 22 | 2 | 24 | 0 | 24 | 0 | 22 | 2 | 20 | 4 | 24 | 0 |
|  | OR (95% CI) | 1.29 (0.29,5.81) | | ND | | ND | | 1.69 (0.37,7.77) | | **3.30 (1.03,10.61)** | | ND | |
| CKD: Chronic Kidney Disease, FLD: Fatty Liver Disease, HF: Heart Failure, LC: Liver Cirrhosis, **Bold: significant confidence intervals** | | | | | | | | | | | | | |

| **Table S3: Sample comorbidities characteristics associated with liver function tests** | | | | | | | | | | | | | | | | | | | | | | | | | | | |
| --- | --- | --- | --- | --- | --- | --- | --- | --- | --- | --- | --- | --- | --- | --- | --- | --- | --- | --- | --- | --- | --- | --- | --- | --- | --- | --- | --- |
| Test |  | Condition | | | | | | | | | | | | | | | | | | | | | | | | | |
|  |  | HTN | | DLD | | HT | | ESRD | | Leukemia | | Cancer | | GERD | | PE | | MI | | Angina | | PVD | | Asthma | | COPD | |
|  |  | N, n | Y, n | N, n | Y, n | N, n | Y, n | N, n | Y, n | N, n | Y, n | N, n | Y, n | N, n | Y, n | N, n | Y, n | N, n | Y, n | N, n | Y, n | N, n | Y, n | N, n | Y, n | N, n | Y, n |
| TBIL | NOR | 148 | 223 | 84 | 287 | 310 | 63 | 370 | 3 | 371 | 2 | 363 | 10 | 362 | 11 | 372 | 1 | 360 | 13 | 367 | 5 | 370 | 3 | 326 | 47 | 370 | 3 |
|  | ABN | 11 | 12 | 5 | 18 | 18 | 5 | 23 | 0 | 23 | 0 | 23 | 0 | 23 | 0 | 22 | 1 | 20 | 3 | 22 | 1 | 23 | 0 | 19 | 4 | 23 | 0 |
|  | *p* | 0.452^a^ | | 0.920^a^ | | 0.568^a^ | | 1^b^ | | 1^b^ | | 1^b^ | | 1^b^ | | 0.113^b^ | | 0.058^b^ | | 0.304^b^ | | 1^b^ | | 0.518^b^ | | 1^b^ | |
|  | OR | 0.72 | | 1.05 | | 1.37 | | ND | | ND | | ND | | ND | | **16.91** | | **4.15** | | 3.34 | | ND | | 1.46 | | ND | |
|  | 95% CI | 0.31,1.68 | | 0.38,2.93 | | 0.49,3.82 | | ND | | ND | | ND | | ND | | **1.03,279.47** | | **1.1,15.77** | | 0.38,29.81 | | ND | | 0.48,4.48 | | ND | |
| Alb | NOR | 149 | 226 | 80 | 295 | 313 | 64 | 375 | 2 | 375 | 2 | 367 | 10 | 366 | 11 | 375 | 2 | 363 | 14 | 370 | 6 | 374 | 3 | 326 | 51 | 374 | 3 |
|  | ABN | 10 | 7 | 5 | 12 | 13 | 4 | 16 | 1 | 17 | 0 | 17 | 0 | 16 | 1 | 17 | 0 | 15 | 2 | 17 | 0 | 17 | 0 | 17 | 0 | 17 | 0 |
|  | *p* | 0.117^a^ | | 0.383^b^ | | 0.510^b^ | | 0.124^b^ | | 1^b^ | | 1^b^ | | 0.415^b^ | | 1^b^ | | 0.147^b^ | | 1^b^ | | 1^b^ | | 0.145^b^ | | 1^b^ | |
|  | OR | 0.46 | | 0.65 | | 1.50 | | **11.72** | | ND | | ND | | 2.08 | | ND | | 3.46 | | ND | | ND | | ND | | ND | |
|  | 95% CI | 0.17,1.24 | | 0.23,1.91 | | 0.48,4.77 | | **1.01,136.09** | | ND | | ND | | 0.26,17.11 | | ND | | 0.72,16.61 | | ND | | ND | | ND | | ND | |
| ALP | NOR | 153 | 231 | 86 | 298 | 320 | 66 | 384 | 2 | 384 | 2 | 377 | 9 | 374 | 12 | 384 | 2 | 371 | 15 | 379 | 6 | 383 | 3 | 336 | 50 | 383 | 3 |
|  | ABN | 8 | 6 | 5 | 9 | 12 | 2 | 13 | 1 | 14 | 0 | 13 | 1 | 14 | 0 | 14 | 0 | 13 | 1 | 14 | 0 | 14 | 0 | 12 | 2 | 14 | 0 |
|  | *p* | 0.195^a^ | | 0.327^b^ | | 1^b^ | | 0.102^b^ | | 1^b^ | | 0.303^b^ | | 1^b^ | | 1^b^ | | 0.441^b^ | | 1^b^ | | 1^b^ | | 0.701^b^ | | 1^b^ | |
|  | OR | 0.50 | | 0.52 | | 0.81 | | **14.77** | | ND | | 3.22 | | ND | | ND | | 1.90 | | ND | | ND | | 1.12 | | ND | |
|  | 95% CI | 0.17,1.46 | | 0.17,1.6 | | 0.18,3.7 | | **1.26,173.45** | | ND | | 0.38,27.36 | | ND | | ND | | 0.24,15.52 | | ND | | ND | | 0.25,5.16 | | ND | |
| GGT | NOR | 35 | 46 | 25 | 56 | 69 | 14 | 83 | 0 | 83 | 0 | 80 | 3 | 80 | 3 | 83 | 0 | 77 | 6 | 80 | 2 | 82 | 1 | 82 | 7 | 82 | 1 |
|  | ABN | 19 | 28 | 14 | 33 | 38 | 9 | 45 | 2 | 47 | 0 | 46 | 1 | 44 | 3 | 47 | 0 | 45 | 2 | 46 | 1 | 47 | 0 | 46 | 9 | 46 | 1 |
|  | *p* | 0.758^a^ | | 0.898^a^ | | 0.743^a^ | | 0.129^b^ | | ND | | 1^b^ | | 0.667^b^ | | ND | | 0.710^b^ | | 1^b^ | | 1^b^ | | 0.074^a^ | | 1^b^ | |
|  | OR | 1.12 | | 1.05 | | 1.17 | | ND | | ND | | 0.58 | | 1.82 | | ND | | 0.57 | | 0.87 | | ND | | 2.29 | | 1.78 | |
|  | 95% CI | 0.54,2.33 | | 0.49,2.31 | | 0.47,2.95 | | ND | | ND | | 0.06,5.74 | | 0.36,9.4 | | ND | | 0.12,2.95 | | 0.08,9.86 | | ND | | 0.81,6.57 | | 0.11,29.18 | |
| PT | NOR | 28 | 42 | 15 | 55 | 59 | 12 | 70 | 1 | 71 | 0 | 69 | 2 | 66 | 5 | 71 | 0 | 65 | 6 | 68 | 3 | 70 | 1 | 57 | 14 | 70 | 1 |
|  | ABN | 10 | 29 | 10 | 29 | 31 | 8 | 37 | 2 | 39 | 0 | 39 | 0 | 39 | 0 | 38 | 1 | 35 | 4 | 37 | 2 | 39 | 0 | 35 | 4 | 38 | 1 |
|  | *p* | 0.132^a^ | | 0.616^a^ | | 0.639^a^ | | 0.286 | | ND | | 0.538^b^ | | 0.159^b^ | | 0.355^b^ | | 0.741^b^ | | 1^b^ | | 1^b^ | | 0.199^a^ | | 1^b^ | |
|  | OR | 1.93 | | 0.79 | | 1.27 | | 3.78 | | ND | | ND | | ND | | ND | | 1.24 | | 1.23 | | ND | | 0.47 | | 1.84 | |
|  | 95% CI | 0.82,4.58 | | 0.32,1.99 | | 0.47,3.44 | | 0.34,43.13 | | ND | | ND | | ND | | ND | | 0.33,4.69 | | 0.2,7.67 | | ND | | 0.15,1.53 | | 0.12,30.29 | |
| INR | NOR | 37 | 66 | 23 | 80 | 87 | 17 | 101 | 3 | 104 | 0 | 102 | 2 | 99 | 5 | 104 | 0 | 95 | 9 | 99 | 5 | 103 | 1 | 86 | 18 | 103 | 1 |
|  | ABN | 1 | 5 | 2 | 4 | 3 | 3 | 6 | 0 | 6 | 0 | 6 | 0 | 6 | 0 | 5 | 1 | 5 | 1 | 6 | 0 | 6 | 0 | 6 | 0 | 5 | 1 |
|  | *p* | 0.663^b^ | | 0.619^b^ | | 0.072^b^ | | 1^b^ | | ND | | 1^b^ | | 1^b^ | | 0.055^b^ | | 0.433^b^ | | 1^b^ | | 1^b^ | | 0.587^b^ | | 0.107^b^ | |
|  | OR | 2.80 | | 0.58 | | 5.12 | | ND | | ND | | ND | | ND | | ND | | 2.11 | | ND | | ND | | ND | | **20.60** | |
|  | 95% CI | 0.32,24.91 | | 0.1,3.35 | | 0.96,27.54 | | ND | | ND | | ND | | ND | | ND | | 0.23,20.1 | | ND | | ND | | ND | | **1.12,379.5** | |
| TP | NOR | 150 | 227 | 83 | 294 | 316 | 63 | 377 | 2 | 378 | 1 | 369 | 10 | 368 | 11 | 378 | 1 | 365 | 14 | 372 | 6 | 376 | 3 | 329 | 50 | 376 | 3 |
|  | ABN | 9 | 6 | 6 | 9 | 11 | 4 | 14 | 1 | 14 | 1 | 15 | 0 | 15 | 0 | 14 | 1 | 13 | 2 | 15 | 0 | 15 | 0 | 15 | 0 | 15 | 0 |
|  | *p* | 0.118^a^ | | 0.118^b^ | | 0.298^b^ | | 0.110^b^ | | 0.075 | | 1^b^ | | 1^b^ | | 0.075^b^ | | 0.119^b^ | | 1^b^ | | 1^b^ | | 0.234^b^ | | 1^b^ | |
|  | OR | 0.44 | | 0.42 | | 1.82 | | **13.46** | | **27.00** | | ND | | ND | | **27.00** | | 4.01 | | ND | | ND | | ND | | ND | |
|  | 95% CI | 0.15,1.26 | | 0.15,1.23 | | 0.57,5.92 | | **1.16,157.45** | | **1.61,454.21** | | ND | | ND | | **1.61,454.21** | | 0.83,19.51 | | ND | | ND | | ND | | ND | |
| ALT | NOR | 36 | 53 | 28 | 61 | 76 | 14 | 89 | 1 | 90 | 90 | 87 | 3 | 86 | 4 | 90 | 0 | 84 | 6 | 88 | 2 | 89 | 1 | 79 | 11 | 88 | 2 |
|  | ABN | 2 | 1 | 1 | 2 | 3 | 0 | 3 | 0 | 3 | 3 | 3 | 0 | 3 | 0 | 3 | 0 | 3 | 0 | 3 | 0 | 3 | 0 | 3 | 0 | 3 | 0 |
|  | *p* | 0.567^b^ | | 1^b^ | | 1^b^ | | 1^b^ | | ND | | 1^b^ | | 1^b^ | | ND | | 1^b^ | | 1^b^ | | 1^b^ | | 1^b^ | | 1^b^ | |
|  | OR | 0.34 | | 0.92 | | ND | | ND | | 1 | | ND | | ND | | ND | | ND | | ND | | ND | | ND | | ND | |
|  | 95% CI | 0.03,3.89 | | 0.08,10.56 | | ND | | ND | | 0.2,5.09 | | ND | | ND | | ND | | ND | | ND | | ND | | ND | | ND | |
| AST | NOR | 129 | 202 | 62 | 269 | 279 | 53 | 330 | 2 | 331 | 1 | 324 | 8 | 323 | 9 | 331 | 1 | 321 | 11 | 326 | 5 | 329 | 3 | 288 | 44 | 331 | 1 |
|  | ABN | 10 | 14 | 5 | 19 | 19 | 5 | 24 | 0 | 23 | 1 | 24 | 0 | 22 | 2 | 24 | 0 | 21 | 3 | 23 | 1 | 24 | 0 | 22 | 2 | 24 | 0 |
|  | *p* | 0.794^a^ | | 0.788^b^ | | 0.566^b^ | | 1^b^ | | 0.130^b^ | | 1^b^ | | 0.165^b^ | | 1^b^ | | 0.060^b^ | | 0.345^b^ | | 1^b^ | | 0.763^b^ | | 1^b^ | |
|  | OR | 0.89 | | 0.88 | | 1.39 | | ND | | 14.39 | | ND | | 3.26 | | ND | | **4.17** | | 2.83 | | ND | | 0.60 | | ND | |
|  | 95% CI | 0.39,2.07 | | 0.32,2.44 | | 0.5,3.88 | | ND | | 0.88,237.59 | | ND | | 0.67,16.03 | | ND | | **1.08,16.1** | | 0.32,25.29 | | ND | | 0.14,2.62 | | ND | |
| ^a^ Pearson’s chi-squared test, ^b^ Fisher’s exact test, ND: not determined, **Bold: significant confidence intervals**. NOR: normal, ABN: abnormal, N: no, Y: yes, HTN: Hypertension, DLD: Dyslipidemia, HT: Hypothyroidism, ESRD: End-stage renal disease, GERD: Gastroesophageal reflux, PE: Pulmonary embolism, MI: Myocardial infarction, PVD: Peripheral vascular disease, COPD: Chronic obstructive pulmonary disease. | | | | | | | | | | | | | | | | | | | | | | | | | | | |

| **Table S4: ORs of sample medication characteristics associated with liver function tests** | | | | | | | | | |
| --- | --- | --- | --- | --- | --- | --- | --- | --- | --- |
| Test |  | T2DM medication | | | | | Other conditions medications | | |
|  |  | Metformin 500 mg^a^ | MET 1000 mg | LIR 5/6 mg | MET 500 mg  + LIR 5/6 mg | MET 1000 mg  + LIR 5/6 mg | None^b^ | APL 100mg | Other^c^ |
| TBIL | Normal | 211 | 105 | 10 | 14 | 10 | 402 | 16 | 9 |
|  | Abnormal | 16 | 5 | 0 | 0 | 0 | 23 | 2 | 2 |
|  | OR (95% CI) | 1.00 (0.49,2.06) | 0.63 (0.23,1.77) | ND | ND | ND | 1.00(0.56,1.82) | 2.19(0.48,10.08) | 3.89(0.8,19.03) |
| Alb | Normal | 220 | 102 | 9 | 12 | 10 | 406 | 15 | 11 |
|  | Abnormal | 8 | 5 | 1 | 2 | 0 | 22 | 3 | 0 |
|  | OR (95% CI) | 1.00 (0.37,2.72) | 1.35 (0.44,4.23) | 3.06 (0.35,27.12) | 4.59 (0.88,23.99) | ND | 1.00(0.55,1.84) | 3.70(1,13.71) | ND |
| ALP | Normal | 219 | 111 | 10 | 14 | 10 | 412 | 17 | 11 |
|  | Abnormal | 8 | 2 | 0 | 0 | 0 | 18 | 1 | 1 |
|  | OR (95% CI) | 1.00 (0.37,2.72) | 0.50 (0.11,2.37) | ND | ND | ND | 1.00(0.52,1.95) | 1.35(0.17,10.69) | 2.09(0.26,17.01) |
| GGT | Normal | 41 | 24 | 2 | 5 | 3 | 89 | 5 | 4 |
|  | Abnormal | 22 | 14 | 2 | 1 | 0 | 49 | 4 | 1 |
|  | OR (95% CI) | 1.00 (0.49,2.09) | 1.09 (0.48,2.52) | 1.87 (0.25,14.16) | 0.38 (0.05,3.4) | ND | 1.00(0.62,1.64) | 1.46(0.38,5.67) | 0.46(0.05,4.18) |
| PT | Normal | 32 | 20 | 3 | 6 | 2 | 89 | 5 | 4 |
|  | Abnormal | 20 | 13 | 0 | 2 | 1 | 49 | 4 | 1 |
|  | OR (95% CI) | 1.00 (0.46,2.21) | 1.04 (0.43,2.55) | ND | 0.54 (0.1,2.91) | 0.80 (0.07,9.41) | 1.00(0.62,1.64) | 1.46(0.38,5.67) | 0.46(0.05,4.18) |
| INR | Normal | 51 | 29 | 3 | 8 | 3 | 111 | 5 | 3 |
|  | Abnormal | 1 | 4 | 0 | 0 | 0 | 3 | 2 | 2 |
|  | OR (95% CI) | 1.00 (0.07,16.43) | 7.04 (0.76,65.97) | ND | ND | ND | 1.00(0.2,5.07) | 14.80**(2.01,109.48)** | 24.67**(2.95,206.58)** |
| TP | Normal | 218 | 107 | 10 | 13 | 9 | 408 | 15 | 10 |
|  | Abnormal | 8 | 3 | 0 | 1 | 0 | 15 | 3 | 1 |
|  | OR (95% CI) | 1.00 (0.37,2.72) | 0.77 (0.2,2.94) | ND | 2.10 (0.25,18.05) | ND | 1.00(0.49,2.08) | 5.44**(1.43,20.83)** | 2.72(0.33,22.65) |
| ALT | Normal | 47 | 26 | 3 | 4 | 1 | 90 | 5 | 5 |
|  | Abnormal | 1 | 1 | 0 | 1 | 0 | 3 | 0 | 0 |
|  | OR (95% CI) | 1.00 (0.07,16.47) | 1.81 (0.11,30.12) | ND | 11.75 (0.62,225.37) | ND | 1.00(0.2,5.09) | ND | ND |
| AST | Normal | 192 | 99 | 4 | 13 | 8 | 346 | 16 | 6 |
|  | Abnormal | 10 | 5 | 3 | 1 | 1 | 22 | 1 | 1 |
|  | OR (95% CI) | 1.00 (0.41,2.46) | 0.97 (0.33,2.92) | 14.40 **(2.84,73.23)** | 1.48 (0.18,12.45) | 2.40 (0.27,21.10) | 1.00(0.55,1.84) | 0.00(0.99,) | 2.63(0.31,22.74) |
| ^a^ Metformin 500mg was used as a reference for OR calculations, ^b^ No additional medication was used as a reference for OR calculations, ^c^ Amiodarone, amoxicillin/clavulanate, and carbamazepine. MET: Metformin, LIR: Liraglutide, APL: Allopurinol. **Bold: significant confidence intervals.** | | | | | | | | | |
